# Gastrointestinal symptoms in patients with mild and severe COVID-19: a scoping review and meta-analysis 

**Published:** 2020

**Authors:** Babak Arjmand, Fatemeh Ghorbani, Mehdi Koushki, Mostafa Rezai-Tavirani

**Affiliations:** 1 *Metabolomics and Genomics Research Center, Endocrinology and Metabolism Molecular* *‐* *Cellular Sciences Institute, Tehran University of Medical Sciences, Tehran, Iran*; 2 *Department of Clinical Biochemistry, Faculty of Medicine, Tehran University of Medical Sciences, Tehran, Iran*; 3 *Department of Clinical Biochemistry, School of Medicine, Zanjan University of Medical Sciences, Zanjan, Iran*; 4 *Proteomics Research Center, Faculty of Paramedical Sciences, Shahid Beheshti University of Medical Sciences, Tehran, Iran *

**Keywords:** COVID-19, Coronavirus, Gastrointestinal symptoms, Digestive symptoms, Meta-analysis

## Abstract

**Aim:**

The current research aimed to analyze and summarize observational studies that compared the incidence of gastrointestinal symptoms in mild and severe COVID-19 infection.

**Background:**

Coronavirus disease 2019 (COVID-19) has been identified as a public health threat worldwide. Previous studies, however, have reported contradictory results of COVID-19-related gastrointestinal symptoms in severe and mild forms.

**Methods:**

A search of Medline, ISI Web of Science, EMBASE, and Cochrane Library databases was conducted for articles published up to May 2020. Data from each study was combined using the random-effects model to calculate odds ratios (ORs) and 95% confidence intervals (95% CIs). Sensitivity was examined by sequentially excluding one study in each turn. Publication bias was evaluated using the Egger’s and Begg’s tests.

**Results:**

Twenty studies (4,265 patients) were reviewed. It was found that the prevalence of diarrhea [OR (0.40), (95% CI 0.91, -2.16), p = 0.03, I2 = 88.1%, PHeterogenity = 0.00)] and nausea and vomiting [OR (0.27), (95% CI 0.07, 1.01), p = 0.05, I2 = 89.3%, PHeterogenity = 0.00)] increased significantly in the severe form compared to the mild form of COVID-19, while abdominal pain and anorexia had no significant increased prevalence in admitted and hospitalized COVID-19 patients. Moreover, COVID-19-related gastrointestinal symptoms were seen in higher rates in males [OR (1.42), (95% CI 1.23, 1.65), p < 0.05, I2= 18.4%, PHeterogenity = 0.23] than in females. No significant publication bias was observed in the meta-analysis. Sensitivity analyses showed a similar effect size while reducing the heterogeneity.

**Conclusion:**

The data provides valuable information for the discovery of prognosis biomarkers to diagnosis more severe disease in the early stages of COVID-19.

## Introduction

 A new coronavirus recently emanated as a pathogen causing pneumonia named severe acute respiratory syndrome coronavirus 2 (SARS-CoV-2), with the disease called coronavirus disease 2019 (COVID-19) (1-3). The disease was initially seen in December 2019 in Wuhan, China, and then quickly spread to many countries. On March 11, 2020, the World Health Organization (WHO) declared it a pandemic (4). Mounting evidence has shown that COVID-19 and SARS-CoV-2 can be transmitted between people through respiratory droplets and close contact with an infected person, posing a major challenge for the global public health system and healthcare settings (5, 6). Currently, there is no vaccine or specific antiviral drug to treat COVID-19 infection. The available antiviral drugs, like HIV-protease inhibitors and nucleoside analogues, are used as supportive therapy to reduce the symptoms and protect organ function (3, 7, 8). The main signs and symptoms of COVID-19 disease include fever, dry cough, tiredness, and breathing difficulty (3, 9, 10). On average, the symptoms of COVID-19 infection appear about 5 days after exposure (11). Gastrointestinal symptoms such as nausea, vomiting, and less commonly diarrhea have also been observed in some COVID-19 patients. Recent studies have reported conflicting results concerning the gastrointestinal symptoms. For example, one study reported digestive symptoms including diarrhea (2.0%) and nausea and vomiting (1.0%) (12). In another research, the patients had clinical characteristics of diarrhea (28%), nausea (9.0%), vomiting (6.0%), and abdominal pain (7.0%) (8). Fan et al. reported the clinical features of diarrhea (4.1%) and nausea and vomiting (2.0%) (13). Pan et al. investigated 99 COVID-19 patients with digestive symptoms. The patients had symptoms of anorexia (83.8%), diarrhea (29.3%), vomiting (8.1%), and abdominal pain (4.0%). In this study, patients with severe infection (n=13) had symptoms of anorexia (100%) and diarrhea (23.1%), whereas patients with moderate symptoms (n=63) displayed anorexia (76.2%) and diarrhea (30.2%) (14). Additionally, Chen et al. recently demonstrated that SARS-CoV-2 RNA is found in the feces of COVID-19 patients (15). Accordingly, more research is needed to determine the relationship between the presence of the virus in the feces samples and gastrointestinal symptoms in order to introduce an indicator for the diagnosis and prognosis of patients. 

The current study aimed to clarify the clinical characteristics and systematically review changes in the gastrointestinal function in SARS-CoV2-infected patients. The results indicate that there is a need to collect sufficient data to aid in the appropriate interpretation of the results. 

## Methods

This meta-analysis was conducted according to the guidelines of the Preferred Reporting Items for Systematic Reviews and Meta-analysis (PRISMA) statement**.**

**Information source and search strategy**

An electronic search of PubMed, Web of Sciences, Scopus, Google Scholar, and Cochran Library was performed for original research articles published up to May 8, 2020, using the following query: (“Digestive Symptoms” OR “Gastrointestinal Symptoms” AND “COVID-19” OR “SARS-Cov19” OR “Severe Acute Respiratory Syndrome Coronavirus” OR “Coronavirus”), (“Diarrhea” AND “COVID-19” OR “SARS-CoV19” OR “Severe Acute Respiratory Syndrome Coronavirus” OR “Coronavirus”), (“Nausea Vomiting” AND “COVID-19” OR “SARS-CoV19” OR “Severe Acute Respiratory Syndrome Coronavirus” OR “Coronavirus”), (“Anorexia” AND “COVID-19” OR “SARS-Cov19” OR “Severe Acute Respiratory Syndrome Coronavirus” OR “Coronavirus”) and (“Abdominal Pain” AND “COVID-19” OR “SARS-CoV19” OR “Severe Acute Respiratory Syndrome Coronavirus” OR “Coronavirus”).

**Selection of studies**

All articles were exported to EndNote software, and duplicated records were removed. There were no restrictions on the language were applied, although all articles used in this meta-analysis were available in English. The reference lists of the included articles were also reviewed manually. Selected articles were screened by title and abstract for eligibility, and the full text was examined by three authors (Nasrin Amiri-Dashatan, Mehdi Koushki, and Masoumeh Farahani) independently. The inclusion criteria included full and observational studies with a retrospective design that focused on COVID-19 patients and published a description of gastrointestinal and digestive symptoms in coronavirus patients.

**Data extraction**

Data was extracted from full-text records using a standard complete extraction sheet. For each included article, data on the first author, publication year, country of study, sample size, mean (SD) age, gender type (female/male), and gastrointestinal symptoms including diarrhea, nausea and vomiting, anorexia and abdominal pain in total in severe and mild groups of COVID-19-affected patients was recorded. 

**Quality of evidence assessment**

The quality of the evidence in the design, analysis, and reporting of outcomes was assessed independently by two reviewers using the Newcastle-Ottawa Quality Assessment Scale (NOS) (16). The studies were assessed across 4 domains: 1) study population selection, 2) exposure, 3) comparability, and 4) outcome. The maximum score for a study was 9 points. Based on the scoring, the studies were classified as I) low quality (0 to 4 points) or II) high quality (5 to 9 points). Disagreements in scoring were resolved through discussion.

**Statistical analysis**

The building of forest plots of binary data on diarrhea, nausea and vomiting, anorexia, and abdominal pain in patients with mild and severe COVID-19 infection was performed using odds ratios (ORs) and 95% confidence intervals (95% CI). In this meta-analysis, the ORs and 95% CI were considered as the effect size to estimate sex (male, female) with severity risk of COVID-19 infection. For each group of variables, a random-effects model was used to calculate ORs of the severity of COVID-19 infection. The heterogeneity among studies was estimated by Q test (significance level at *p* < 0.1) and I^2^ statistics. The I^2^ statistics were characterized by the percentage of the total variation in effect size that can be associated with heterogeneity. Values greater than 50% and 70% were considered as moderate to high heterogeneity, respectively. Sensitivity was analyzed by sequentially excluding one study in each turn to evaluate the robustness of the results. The Begg’s rank correlation test and Egger’s regression asymmetry test were also applied to evaluate the potential publication bias obtained by the funnel plot (17, 18). Trim-and-fill analysis was used to regulate any significant publication bias detected. Lastly, a restricted maximum likelihood-based random effects meta-regression analysis was performed to evaluate the relationship between the confounder variable of gender on overall effect size. The CMA (comprehensive meta-analysis) V2 software (Biostat, NJ, USA) (19) was used for this meta-analysis. A *p-*value < 0.05 was considered statistically significant. 

## Results

**Overview of included studies**

**Figure 1:**
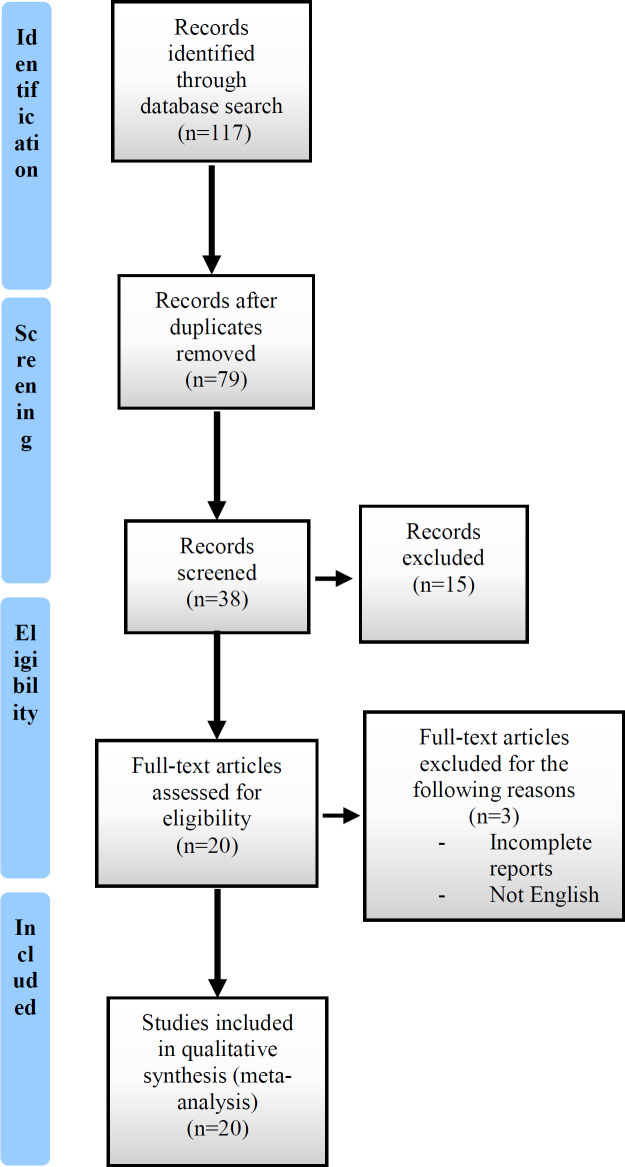
Flow chart of study selection for meta-analysis

The electronic databases were searched for articles published through May 8, 2020. A total of 117 articles were initially found, including 114 English and 3 Chinese articles, respectively. Of these, 79 records were excluded from our analysis due to duplication or because they were reviews or letters. Of the remaining 38 articles, 15 articles were rejected for lack of enough relevant information, and 3 articles were removed due to incomplete reports and not published in the English language. Finally, 20 eligible articles were included in this study. The outline of the flowchart of study selection is summarized in Figure 1. 

**Characteristics and quality of included studies**

The baseline characteristics of the 20 studies are summarized in Table 1.The cases evaluated by the studies included in this meta-analysis comprised patients with mild and severe COVID-19 infections. 

**Figure 2:**
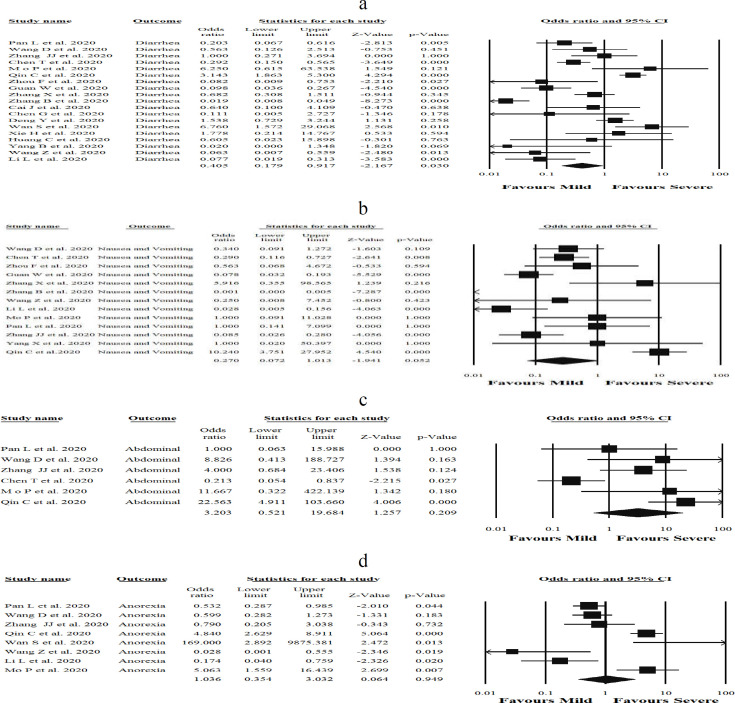
Forest plots detailing odds ratio (OR) and 95% confidence intervals of the incidence of COVID-19-related gastrointestinal symptoms of a) diarrhea, b) nausea and vomiting, c) abdominal pain, and d) anorexia in admitted and hospitalized patients. Meta-analysis was performed using a random-effects model

**Table 1:** Baseline characteristics of the included studies

Athour's name/ Country (ref)	Samples (N)	Sex (M/ F)	Age (year)Mean ± SD	Digestive symptom				Study Quality
				abdominal PainN (%)	AnorexiaN (%)	DiarrheaN (%)	Nausea & Vomiting N (%)	
Zhou F et al. (2020)/ China (20)	Total: 191Mild: 137Severe: 54	Total: 119/72Mild: 81/56 Severe: 38/16	Total: 56.25 ± 3.5Mild: 51.75 ± 2.16Severe: 69.25 ± 3.25	-	-	Total: 9 (5)Mild: 7 (5)Severe: 2 (4)	Total: 7 (4)Mild: 4 (3)Severe: 3 (6)	8
Huang C et al. (2020)/ China (9)	Total: 38Mild: 25Severe: 13	Total: 30/11Mild: 19/9Severe: 11/2	Total: 49.2 ± 4.25Mild: 49.12 ± 4.12Severe: 50 ± 5	-	-	Total: 25 (3) Mild: 1 (12)Severe: 0 (0)	-	6
Pan L et al. (2020)/ China (14)	Total: 99Mild: 64Severe: 35	Total: 54/45 Mild: 33/31Severe: 21/14	Total: 54.6 ± 16.1	Total: 4 (4)Mild: 2 (3.2) Severe: 2 (9.1)	Total: 83 (83.8)Mild: 48 (76.2)Severe: 35 (100)	Total: 29 (29.3)Mild:: 20 (31.2)Severe: 9 (25.7)	Total: 8 (8.1)Mild: 4 (6.4)Severe: 4 (11.4)	6
Guan W et al. (2020)/ China (21)	Total: 1096Mild: 923Severe: 173	Total:637/ 459Mild: 537/3861Severe: 100/73	Total: 46.75 ± 4.16Mild: 45.25 ± 3.83Severe: 52.25 ± 4.16	-	-	Total:42 (3.8)Mild: 32 (3.5)Severe: 10 (5.8)	Total:55 (5)Mild: 43 (4.6)Severe: 12 (6.9 )	5
Wang D et al. (2020)/China (22)	Total: 138Mild: 102Severe: 36	Total: 75/63Mild: 53/49 Severe: 22/14	Total: 55.5 ± 4.33Mild: 50.25 ± 4.16Severe: 66.75 ± 5.25	Total: 3 (2.2)Mild: 0 (0)Severe: 3 (8.3)	Total: 55 (39.9)Mild: 31 (30.4)Severe: 24 (66.7)	Total: 14 (10.1)Mild: 8 (7.8)Severe: 6 (16.7)	Total: 19 (13.7)Mild: 12 (11.76)Severe: 7 (19.44)	8
Zhang JJ et al. (2020)/ China (23)	Total: 140Mild: 82Severe: 58	Total: 71/69Mild: 38/44Severe: 33/25	Total: 56.6 ± 10.33Mild: 51.75 ± 8.66Severe: 60 ± 15.5	Total: 8 (5.8)Mild: 2 (5.8)Severe: 6 (10.2)	Total:17 (12.2)Mild: 9 (11)Severe: 8 (14)	Total: 18 (12.9)Mild: 9 (11)Severe: 9 (15.8)	Total:31 (22.3)Mild: 24 (29.2)Severe: 7 (12.2)	6
Zhang X et al. (2020)/ China (24)	Total: 645Mild: 72Severe: 573	Mild: 33/39Severe: 295/278	Mild: 34.9 (14.2)Severe: 46.65 ± 13.82	-	-	Mild: 8 (11.1)Severe: 45 (7.9)	Mild: 0 (0)Severe: 22 (3.8)	7
Zhang et al. (2020)/ China (25)	Severe: 82	Severe: 54/28	Severe: 72.5 ± 2.5	-	-	Severe: 10 (12.2)	Severe: 2 (2.3)	4
Cai J et al. (2020)/ China (26)	Total:298Mild: 240Severe: 58	Total: 145/153Mild: 106/134Severe: 39/19	Total: 47.2 ± 4.6Mild: 42.25 ± 4.16Severe: 61.75 ± 2.5	-	-	Total: 9 (3.02)Mild: 5 (2.08)Severe: 4 (6.9)	-	5
Chen G et al. (2020)/ China (27)	Total:21Mild: 10Severe: 11	Total:17/4Mild: 7/3 Severe: 10/1	Total:56.3 ± 14.3Mild: 51.4 ± 13.7Severe: 63.9 ± 9.6	-	-	Total: 4 (20)Mild: 3 (10)Severe: 1 (10)	-	7
Chen T et al. (2020)/ China (8)	Total:274Mild: 161Severe: 113	Total:171/102Mild: 88/73Severe: 83/30	Total:59.5 ± 4.33Mild: 51.25 ± 4.83Severe: 68.75 ± 2.5	Total: 19 (7)Mild: 13 (8)Severe: 6 (5)	-	Total: 77 (28)Mild: 50 (31)Severe: 27 (24)	Total: 40 (15)Mild: 26 (16)Severe: 14 (12)	6
Deng Y et al. (2020)/ China (28)	Mild: 116Severe: 109	Mild: 51/73Severe: 65/36	Mild: 42.5 ± 4Severe: 68.5 ± 2	-	-	Mild: 14 (12.1)Severe: 19 (17.4)	-	6
M o P et al. (2020)/ China (29)	Total: 155Mild: 70Severe: 85	Total: 86/69Mild: 31/39Severe: 55/30	Total: 54 ± 4Mild: 45.75 ± 3.5Severe: 60.75 ± 3.16	Total: 3 (1.9)Mild: 0 (0)Severe: 2 (2.4)	Total: 26 (31.7)Mild: 8 (18.2)Severe: 18 (47.4)	Total: 7 (4.5)Mild: 2 (2.9 )Severe: 5 (5.9 )	Total:6 (7.4)Mild: 2 (4.5)Severe: 2 (5.3)	7
Wan S et al. (2020)/ China (30)	Total: 135Mild: 95Severe: 40	Total: 72/63 Mild: 52/43Severe: 21/19	Total: 46.25 ± 3.16Mild: 42.5 ± 2.66Severe: 59.25 ± 3.5	-	Total: 6 (4.4)Mild:: 0 (0)Severe: 6 (15)	Total:18 (13.1)Mild: 5 (5.3)Severe: 13 (32.5)	-	7
Xie H et al. (2020)/ China (31)	Total: 79Mild: 51Severe: 28	Total: 44/34Mild: 26/25Severe: 18/10	Total: 58.5 ± 3Mild: 57.5 ± 5Severe: 60.82 ± 4.32	-	-	Total:7 (8.9)Mild: 3 (5.9)Severe: 4 (14.3)	-	6
Yang X et al. (2020)/ China (32)	Total: 52Mild: 20Severe: 32	Total: 35/17Mild: 14/6Severe: 21/11	Total: 59.7 ± 13.3Mild: 51.9 ± 12.9Severe: 64.6 ± 11.2	-	-	-	Total:2 (4)Mild: 1 (5)Severe: 1 (3)	4
Lin L et al. (2020)/ China (33)	Total: 58 Mild: 47 Severe: 11	Total: 27/ 31	Total: 48 ± 17.1	-	Total: 17 (17.9)Mild:: 12 (12.6)Severe: 5 (5.3)	Total: 23 (24.2)Mild:: 18 (18.9)Severe: 5 (5.3)	Total: 21(22.1)Mild:: 18 (18.9)Severe: 3 (3.2)	6
Qin C et al. (2020)/ China (34)	Total: 452 Mild: 166 Severe: 286	Total: 235/217 Mild: 80/86 Severe: 155/131	Total: 57.5 ± 3.33 Mild: 52.3 ± 3.45 Severe: 60.5 ± 3.0	Total: 23 (5.0) Mild: 4 (2.4) Severe: 19 (6.6)	Total: 92 (21) Mild: 30 (18.1) Severe: 66 (23.1)	Total: 122 (26.7) Mild: 44 (26.5) Severe: 78 (27.3)	Total: 42 (9.2) Mild: 10 (6.0) Severe: 32 (11.2)	7
Yang B et al. (2020)/ China (35)	Total: 18 Mild: 12 Severe: 6	Total: 9/9 Mild: 7/5 Severe: 2/4	Total: 49.5 ± 10.5 Mild: 40.25 ± 6.25 Severe: 58 ± 6.5	-	-	Total: 3 (17.0) Mild: 3 (25.0) Severe: 0 (0.0)	-	5
Wang Z et al. (2020)/ China (36)	Total: 69 Mild: 55 Severe: 14	Total: 32/37 Mild: 25/30 Severe: 7/7	Total: 45.25 ± 4.5 Mild: 39.25 ± 4.75 Severe: 70 ± 3.75	-	Total: 7 (10.0) Mild: 6 (11.0) Severe: 1 (7.0)	Total: 10 (14.0) Mild: 8 (15.0) Severe: 2 (14.0)	Total: 3 (4.0) Mild: 2 (4.0) Severe: 1 (7.0)	7

M: Male; F: Female

A total of 1817 and 2448 COVID-19 patients with severe and mild phenotypes, respectively, were evaluated in this meta-analysis. All included articles were published in 2020 by China and were observational in design. COVID-19 was diagnosed based on real-time RT-PCR in all studies. Only 4 (26.6%) of the included studies evaluated all gastrointestinal symptoms including diarrhea, nausea and vomiting, anorexia, and abdominal pain. The quality of the studies is presented in Table 1, indicating the overall admissible quality of the included studies. As can be seen, 2 and 18 articles were considered as low and moderate to high quality, respectively.

**Gastrointestinal symptoms in severe and mild COVID-19 infection**

**Figure 3:**
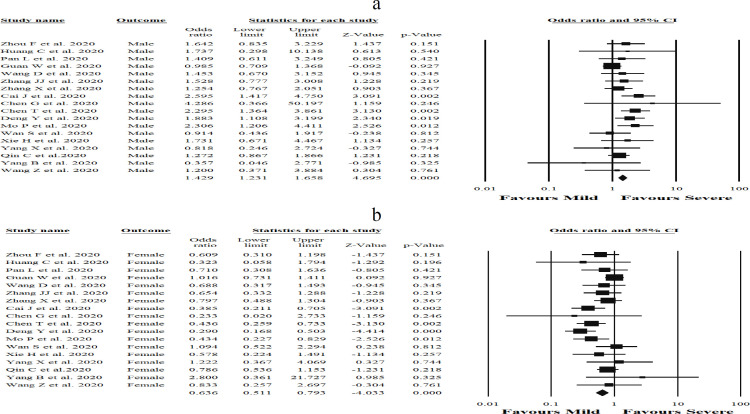
Forest plots assessing odds ratio (OR) and 95% confidence intervals between genders, a) male and b) female, and risk of increased incidence of COVID-19-related gastrointestinal symptoms in admitted and hospitalized patients. Meta-analysis was performed using a random-effects model

The current study evaluated whether patients with gastrointestinal symptoms including abdominal pain, anorexia, nausea and vomiting, or diarrhea may be at elevated risk for the severe form of COVID-19, and the results are shown in Fig. 2. Diarrhea [OR (0.40), (95% CI 0.91, -2.16), *p *= 0.03, I^2^ = 88.1%, *P*_Heterogenity_ = 0.00)] (Fig. 2a) and nausea and vomiting [OR (0.27), (95% CI 0.07, 1.01), *p *= 0.05, I^2^ = 89.3%, *P*_Heterogenity_ = 0.00)] (Figure. 2b) were found to be significantly associated with severity of COVID-19, while abdominal pain [OR (3.2), (95% CI 0.52, 19.6), *p *= 0.20, I^2^ = 77.7%, *P*_Heterogenity_ = 0.00)] (Fig. 2c), and anorexia [OR (1.03), (95% CI 0.35, 3.03), *p *= 0.94, I^2^ = 87.1%, *P*_Heterogenity_ = 0.00)] (Fig. 2d) were not significantly associated with increased COVID-19 severity. On the other hand, the summary OR for male patients with severe COVID-19 infection was [OR (1.42), (95% CI 1.23, 1.65), *p *< 0.05, I^2^= 18.4%, *P*_Heterogenity_ = 0.23] (Fig. 3a) compared to females [OR (0.63), (95% CI 0.79, -4.03), *p *< 0.05, I^2^= 43.3%, *P*_Heterogenity_ = 0.02] (Fig. 3b). Strikingly, it was found that males are associated with a near 2.5-fold increase in odds of having severe COVID-19. Overall, these findings confirm that gender is an important factor that can affect the severity of COVID-19 infection. Statistical significant heterogeneity was observed within the studies included in the present meta-analysis. Sensitivity was evaluated using the leave-one-out method. Interestingly, when every single low-quality study was removed, the overall effect size of the variables was not changed. In addition, the overall effect size remained significantly higher in patients with severe COVID-19 following the removal of studies with larger sample sizes, which was nearly 49% of the pooled sample size. 

**Figure 4:**
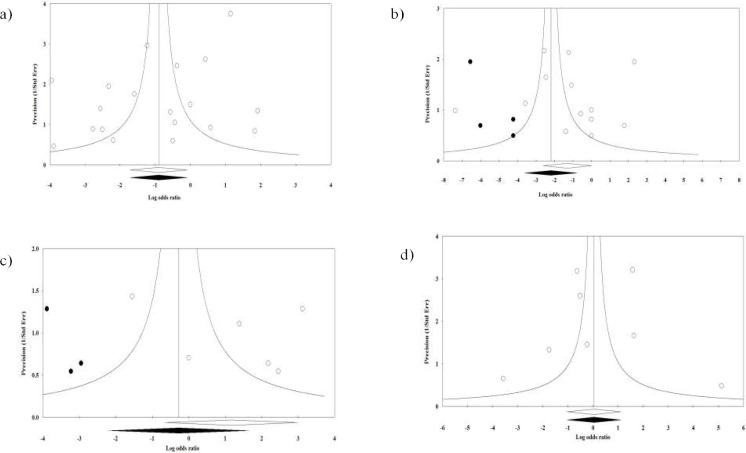
Random effects funnel plots detailing publication bias in the studies investigating the gastrointestinal symptoms of a) diarrhea, b) nausea and vomiting, c) abdominal pain, and d) anorexia in severe and mild forms of COVID-19 in admitted and hospitalized patients after trimming and filling. Open circles represent observed published studies; closed circles represent imputed unpublished studies

**Publication bias**

The Begg’s rank correlation tests (diarrhea; Kendall’s tau with continuity correction = 0.00, Z=0.00, 2-tailed *p*-value= 1.00), (nausea and vomiting; Kendall’s tau with continuity correction = 0.16, Z = 0.79, 2-tailed *p-*value = 0.42), (abdominal pain; Kendall’s tau with continuity correction = 0.26, Z = 0.75, 2-tailed *p-*value = 0.45) and (anorexia; Kendall’s tau with continuity correction = -0.03, Z = 0.12, 2-tailed *p*-value = 0.90) and the Egger’s linear regression tests (diarrhea; *p* = 0.25, nausea and vomiting; *p* = 0.87, abdominal pain; *p* = 0.57, and anorexia; *p* = 0.80) were not statistically significant. Also, the funnel plot of the study precision (inverse standard error) per effect size (Log OR) was symmetric and indicated no potential publication bias in reporting the primary outcomes in patients with severe or mild COVID-19 (Fig. 4). It was observed that trim-and-fill correction was similar in the values of the observed studies and imputed missing studies in those reporting the primary outcomes of diarrhea and anorexia in patients with COVID-19. As for abdominal pain in patients with COVID-19, trim-and-fill correction imputed 3 possibly missing studies, resulting in a correct effect size of (OR: 0.75) (95% CI 0.11, 5.16). As reported by “fail safe N”, 4 theoretically missing studies were needed to import the *p*-value to lower than 0.05. In addition, in presenting studies of nausea or vomiting in patients with severe and mild COVID-19 in which there was no funnel plot asymmetry, trim-and-fill correction imputed 4 potentially missing studies which led to a correct effect size of (OR: 0.11) (95% CI 0.02, 0.46). The “fail safe N” method showed that 99 theoretically missing studies were needed to create a significant effect. 

**Restricted maximum likelihood meta-regression**

**Figure 5:**
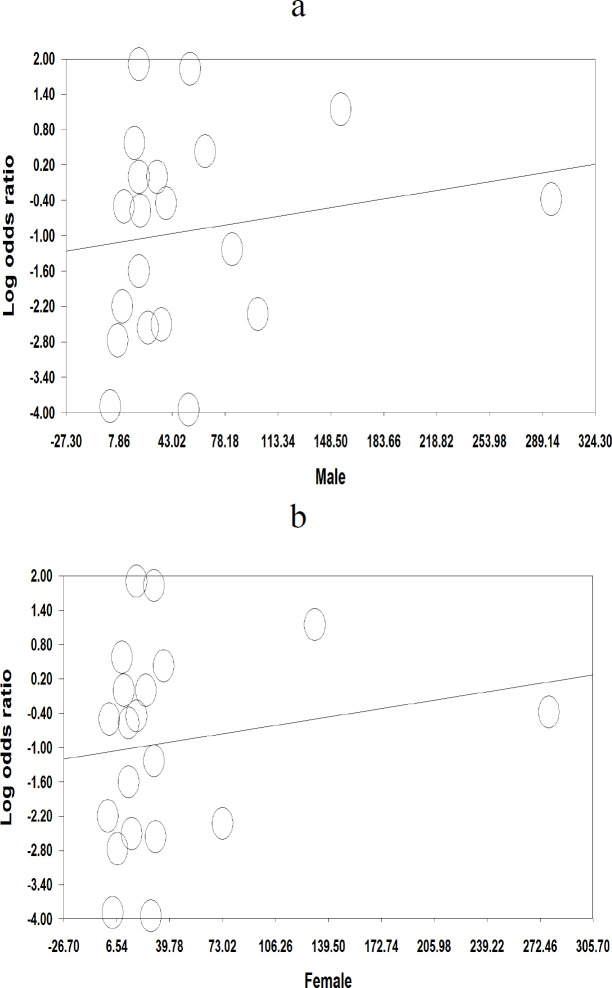
Random effects meta-regression plots of the association between the prevalence of gastrointestinal symptoms in COVID-19 patients with the variable of gender. The size of each circle is inversely proportional to the variance of change. Meta-analysis was performed using a random-effects model

Random effects meta-regression was performed to assess whether the prevalence of gastrointestinal symptoms was associated with gender. The results indicated that males significantly influence the pooled effect size of the incidence of gastrointestinal symptoms compared to females (Figure 5).

## Discussion

In the current study, all articles reporting gastrointestinal symptoms among patients with mild and severe COVID-19 infection published up to May 8, 2020 were reviewed. All of the included studies came from China. COVID-19 patients are still mainly known by the respiratory system, but evidence of other organ involvement, such as gastrointestinal (GI) symptoms, has been reported. In the present study, a pooled analysis of studies including COVID-19-related gastrointestinal symptoms through a systematic review and meta-analysis demonstrated that diarrhea and nausea and vomiting were found to be associated with an increased risk of severe COVID-9, whereas abdominal pain and anorexia had no significant increased prevalence in severe cases of COVID-19 compared to milder forms. Based on these results, it was hypothesized that diarrhea and nausea and vomiting in severe cases are promoted through direct injury to the intestinal mucosa following virus accumulation in the gut. The results revealed that the variable of gender has a significant effect on the severity of COVID-19, a finding which was confirmed by meta-regression analysis. The prevalence of COVID-19-related gastrointestinal symptoms was significantly increased in males compared to females. The current results support that the prevalence of diarrhea and nausea and vomiting in male patients referring to a hospital should be considered to better understand how COVID-19 affects the gastrointestinal tract. 

The frequency of GI symptoms varied widely, from 3.0% to 39.6%, in the published papers (37). A recent study reported that nearly one-half of COVID-19 patients showed GI symptoms as their main complaint, and among the GI symptoms, diarrhea was the most commonly reported symptom. Hence, the diagnosis of COVID-19 in patients with GI symptoms is delayed, which can lead to exacerbation of the disease (14). Several recent studies have reported patients initially presenting with GI symptoms only; therefore, medical staff should be vigilant in examining clients presenting with GI symptoms. There are several reasons for the appearance of digestive symptoms among COVID-19 patients. The direct interacting and binding of the virus to the host cells and inflammatory chain response are two main points in this regard. The results of previous studies have shown that angiotensin-converting enzyme 2 (ACE2) is the main receptor to the entry of COVID-19 into the cells. Although cholangiocytes, type II alveolar, and kidney cells have been introduced as cells that express ACE2, the expression of this receptor in the mucosa of the oral cavity and GI tract has recently been reported (38). On the other hand, some studies isolated the viral from stool samples of COVID-19 patients. Therefore, based on the available evidence, the appearance of GI symptoms in COVID-19 patients can be the result of a direct attack of the virus on the cells of the GI tract and their entry through the ACE2 receptor, altering the natural flora of the intestines, and indirectly through systemic inflammation (39). These possible mechanisms are hypothesized, and further investigation is required to explore the underlining mechanisms for GI damage in COVID-19 patients. 

Diarrhea and vomiting were found to have a relationship with the severity of COVID-19 infection. It is thought that diarrhea and vomiting may possibly be related to the amount of the virus in the intestine, which may lead to increased severity of illness observed to be related to a high viral load. These results are in agreement with the results of previous studies (24, 28). In contrast, however, the relationship between abdominal pain and anorexia with COVID-19 severity has not been shown in this study, which can be explained by the small number of examined samples and studies reporting these variables. 

This meta-analysis has several limitations: 1) Most of the included studies were performed in one specified geographical region of the world; 2) Clinical trials are needed to focus more intently on the better identification of diagnostic and prognostic indicators in COVID-19 patients with digestive symptoms; 3) Finally, given the incidence of digestive symptoms in the early stages of infection, most of the included studies were conducted in hospitalized patients after admission. 

In conclusion, this meta-analysis noted that of 4,265 patients with confirmed COVID-19 in China, diarrhea, nausea and vomiting were associated with severe outcomes from COVID-19 infection, whereas abdominal pain and anorexia had no significant increased prevalence in hospitalized patients with severe COVID-19. We suggest that gastrointestinal symptoms of diarrhea, nausea and vomiting may be used as clinical prognosticators of severe COVID-19. Such consideration can lead to a more appropriate treatment protocol for the treatment of people with more severe disease.
